# STAT3 phosphorylation at Ser727 and Tyr705 differentially regulates the EMT–MET switch and cancer metastasis

**DOI:** 10.1038/s41388-020-01566-8

**Published:** 2020-12-01

**Authors:** Wei-Hsin Lin, Yi-Wen Chang, Min-Xiang Hong, Te-Cheng Hsu, Ko-Chuan Lee, Che Lin, Jia-Lin Lee

**Affiliations:** 1 Institute of Molecular and Cellular Biology, National Tsing Hua University, Hsinchu, 30013 Taiwan; 2 Department of Orthopedic Surgery, National Taiwan University Hospital, Taipei, 100225 Taiwan; 3 Institute of Communications Engineering, National Tsing Hua University, Hsinchu, 30013 Taiwan; 4 Department of Electrical Engineering and Graduate Institute of Communication Engineering, National Taiwan University, Taipei, 10617 Taiwan; 5 Department of Medical Science, National Tsing Hua University, Hsinchu, 30013 Taiwan

**Keywords:** Cancer microenvironment, Cancer stem cells

## Abstract

Epithelial–mesenchymal transition (EMT)/mesenchymal–epithelial transition (MET) processes are proposed to be a driving force of cancer metastasis. By studying metastasis in bone marrow-derived mesenchymal stem cell (BM-MSC)-driven lung cancer models, microarray time-series data analysis by systems biology approaches revealed BM-MSC-induced signaling triggers early dissemination of CD133^+^/CD83^+^ cancer stem cells (CSCs) from primary sites shortly after STAT3 activation but promotes proliferation towards secondary sites. The switch from migration to proliferation was regulated by BM-MSC-secreted LIF and activated LIFR/p-ERK/pS727-STAT3 signaling to promote early disseminated cancer cells MET and premetastatic niche formation. Then, tumor-tropic BM-MSCs circulated to primary sites and triggered CD151^+^/CD38^+^ cells acquiring EMT-associated CSC properties through IL6R/pY705-STAT3 signaling to promote tumor initiation and were also attracted by and migrated towards the premetastatic niche. In summary, STAT3 phosphorylation at tyrosine 705 and serine 727 differentially regulates the EMT–MET switch within the distinct molecular subtypes of CSCs to complete the metastatic process.

## Introduction

Metastasis, which causes more than 90% of cancer-related deaths, is a multistage process during which malignant cells spread from the primary tumor into distant organs. The “early dissemination” definition was refined by Husemann et al. [1] when they found that early disseminated cancer cells (eDCCs) originate at times when lesions are only defined in situ by light microscopy (a clinically latent stage of hidden cancer spread). Cancer of unknown primary is a relatively frequent event in solid cancers where metastases develop without the presence of an obvious primary tumor mass that evolved to become invasive [2]. In fact, at the time of diagnosis, cancer cell dissemination has occurred in >50% of patients [3]. In addition, eDCCs detected in patients before the manifestation of cancer metastasis contain fewer genetic abnormalities than primary tumors in breast cancer [4], pancreatic cancer [5], and melanoma [6] models, indicate that dissemination might occur during the early stages of tumor evolution [1]. However, the mechanisms that might allow eDCCs to complete all steps of metastasis are unknown.

The current understanding of epithelial carcinoma metastasis highlights the importance of epithelial–mesenchymal transition (EMT) in equipping tumor cells with motility and invasiveness [7]. Earlier studies showed that cancer cells acquired stemness after undergoing EMT [8]. Upon histopathological examination, however, cells in metastatic nodules often resemble those in the primary site. This observation suggests that once migrating cancer stem cells (CSCs) reach a suitable distant site, they usually need to revert to the epithelial phenotype through mesenchymal–epithelial transition (MET) to reacquire their proliferative ability and eventually form metastatic colonies. Epithelial plasticity, defined as the transition between the epithelial and mesenchymal phenotypes, is therefore crucial in the initiation and establishment of cancer metastasis [9]. However, to date, the mechanism by which tumor cells commence and resolve the EMT–MET program as they proceed through the metastatic cascade remains incompletely understood.

The skeleton is the third most common site for cancer metastasis after the lung and liver. The detection of eDCCs in bone marrow is associated with poor prognosis [10]. DCC detection is not only a predictor for metastatic relapse in bone but also for local relapse [11], supporting recent experimental data that metastatic cancer cells may recirculate from bone marrow back to the primary site where they might contribute to local relapse and even primary tumor growth [12]. Thus, bone marrow might be a reservoir for metastatic cancer cells in which they may survive for an extended period of time [13]. Multiple types of cells reside in the bone stroma, each of which performs different physiological functions. Among these cells, bone marrow-derived mesenchymal stem cells (BM-MSCs) are principally responsible for repairing damaged tissues, such as fractures or wounds [14]. In the bone marrow, MSCs may be involved in establishing tumor dormancy. Metastatic tumor cells come into contact with local MSCs after they reach distant metastatic sites such as the BM. These native BM-MSCs then constitute part of the metastatic niche. In addition, BM-MSCs have been shown to migrate towards solid tumors and become incorporated into the primary tumor stroma [15]. Unfortunately, our knowledge of the role of BM-MSCs in cancer development is cripplingly limited.

We therefore addressed the issue of lung-cancer-cell dissemination soon after/before cancer initiation and investigated whether mechanisms exist that reduce metastatic seeding from primary cancers. We may also be able to understand how eDCCs found metastasis directly and/or through the preparation of eDCC-mediated premetastatic niches for later arriving metastatic cancer cells to colonize target organs. In summary, we report on a mechanism involving BM-MSCs, the EMT–MET program and STAT3 signaling that reconciles early and late dissemination models. LIF/pS727-STAT3-elicited MET followed by IL6/pY705-STAT3-elicited EMT regulated by BM-MSCs is required for CSC heterogeneity and cancer metastasis. These findings might inform on better ways to target eDCCs in all their forms to prevent metastasis.

## Results

### Isolation and characterization of human BM-MSCs

MSCs were isolated from BM collected during orthopedic arthroplasty procedures in patients. After passaging, these cells displayed consistent morphology (Supplementary Fig. 1a, b) and stably expressed several markers commonly used to characterize MSCs, such as CD44, CD105, CD45, and CD133 (Supplementary Fig. 1c, d). Significant elevation of cytokines concentrations, such as IL-6, LIF, CCL2, and IL-8, was observed in a human cytokine antibody array after culture with BM-MSCs (Supplementary Fig. 1e), indicating that BM-MSCs can secrete these cytokines.

#### Identification of a gene expression signature linked to early dissemination

The CSC theory posits that CSCs mediate metastasis, are resistant to chemotherapy, and contribute to relapse. Liu et al. demonstrated that breast CSCs can exist in distinct mesenchymal-like (EMT) and epithelial-like (MET) states [16]. This group showed that mesenchymal-like breast CSCs, characterized as CD24^−^/CD44^+^, are primarily quiescent, whereas epithelial-like breast CSCs express aldehyde dehydrogenase and can proliferate. It was proposed that the phenotypic plasticity of CSCs in transitioning between the epithelial and mesenchymal states endows them with the capacity for tissue invasion, dissemination, and growth at metastatic sites. We hypothesize that CSCs not only exist in different states but also respond differently to BM-MSCs based on their cellular context.

**Fig. 1:**
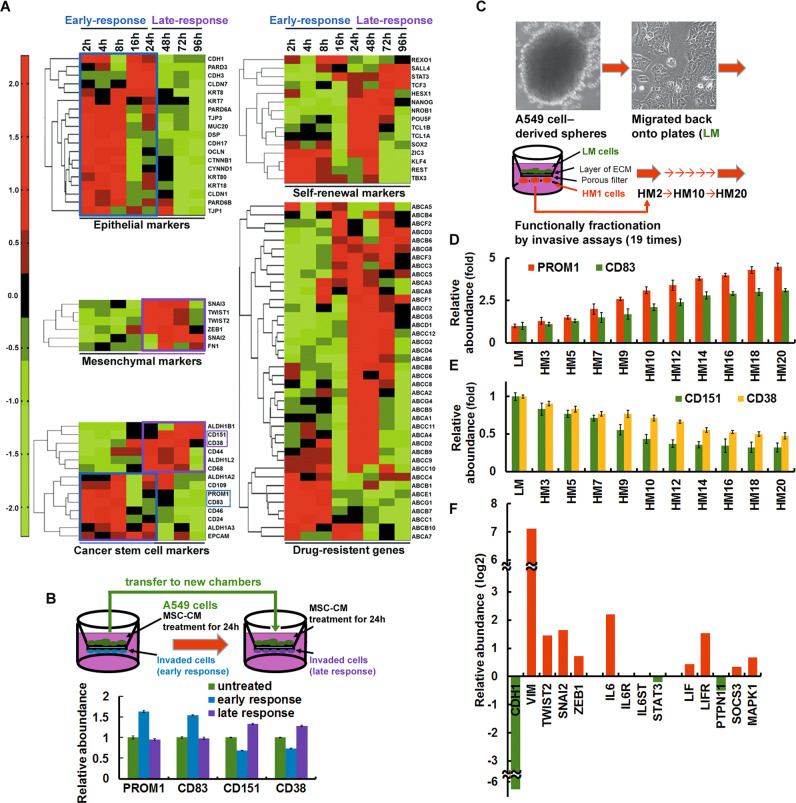
Identification of a gene expression signature linked to early dissemination. **A** Microarray time-series data analysis was performed on cells after treatment with BM-MSC-CM at 9 time points, from 0 to 96 h. Representative clusters of the indicated genes are shown as heatmaps, with red indicating increased expression and green indicating decreased expression, as indicated by the color intensity scale shown below each heatmap. **B** Functional fractionation of A549 cells via invasion assays. The percentage of cells expressing surface markers was determined by flow cytometry (CD133^+^/CD83^+^ for early-response cells; CD151^+^/CD38^+^ for late-response cells). **C**–**E** Functional fractionation of cancer cells via serial invasion assays. Cells were serially selected through 5 (HM5), 10 (HM10), or 20 (HM20) Matrigel invasion assays. The percentage of cells expressing surface markers was determined by QPCR (CD133^+^/CD83^+^ in **D**; CD151^+^/CD38^+^ in **E**). **F** QPCR showing the mRNA expression levels of STAT3 signaling-related genes. HM20 cells are compared to LM cells.

In order to identify a gene expression signature linked to early dissemination induced by BM-MSCs, we performed global gene expression analysis of BM-MSC-CM treated human lung cancer A549 cells for 2, 4, 8, 16, 24, 48, 72, and 96 h as indicated. The microarray data on both early- (2–24 h, left panel) and late-response (24–96 h, right panel) subgroups provided evidence that signal transduction is differentially activated depending on the BM-MSC-CM treatment time (Fig. 1A). More importantly, after treatment with BM-MSC-CM, many CSC marker [*PROM1* (CD133) and *CD83*] and epithelial marker (*CDH1*) genes were substantially upregulated in early-response cells (2–24 h, left panel), whereas other CSC marker (*CD151* and *CD38*) and mesenchymal marker (*Twist1* and *FN1*) genes were upregulated in late-response cells (24–96 h, right panel) (Fig. 1A). Consistent with the results of Fig. 1A, similar results were shown in human lung cancer H322 cells. To confirm the results of microarray time-series data analysis, A549 cells were placed in the upper chamber of an invasion assay kit that contained conditioned medium from BM-MSCs (BM-MSC-CM) (Fig. 1B). Cells that invaded through the membrane in the chamber within 24 h of BM-MSC-CM treatment were collected and identified as early-response cells. Cells that remained in the chamber after 24 h were subsequently transferred into a new chamber, where BM-MSC-CM treatment was continued. The cells that invaded through the membrane in the new chamber after another 24 h of BM-MSC-CM treatment were identified as late-response cells. Consistent with the results of Fig. 1A, early-response cells were CD133^+^/CD83^+^, and late-response cells were CD151^+^/CD38^+^ (Fig. 1B). In order to fractionate early-response cells and late-response cells, functional fractionation was performed on A549 cancer cells by subjecting them to an invasion assay [17]. The cells that remained in situ were designated LM cells, and the cells that migrated through the membrane were designated HM cells. The HM cell phenotype was further enhanced and stabilized through 19 rounds of invasion assays, with the invaded cells harvested from each subsequent round of selection designated HM2 to HM20 cells (Fig. 1C). HM20 cells stably expressed CD133 and CD83, while LM cells were CD151^+^/CD38^+^ (Fig. 1D, E). These findings indicated that early-response cells were analogous to HM20 cells and that late-response cells were equivalent to LM cells. Compared to late-response (LM) cells, the early-response (HM20) cells exhibited an enhanced mesenchymal phenotype, as demonstrated by a decrease in the level of the epithelial marker E-cadherin (*CDH1*) and an increase in the levels of mesenchymal markers (e.g., Vimentin, Twist2, and Snail2). We also observed an increase in LIF and LIFR expression in HM20 cells (Fig. 1F). CL1-5 is CL1-0-derived human lung adenocarcinoma cell line with different degrees of invasiveness; CL1-5 cells are more invasive than CL1-0 cells (kindly provided by Dr. Pan-Chyr Yang at National Taiwan University) [18]. Consistent with the results of Fig. 1D–F, similar results were shown in CL1-0 and CL1-5 cells (Supplementary Fig. 2a–c). Functional fractionation was also performed on A549, H1299, H460, and H322 lung cancer cells by subjecting them to an invasion assay. The cells that remained in situ were designated IV1 cells. The IV1 cell phenotype was further enhanced and stabilized through 19 rounds of invasion assays (under BM-MSC-CM stimulation), with the invaded cells harvested from each subsequent round of selection designated IV2 to IV20 cells. IV1 derived from A549, H1299, H460, and H322 cells stably expressed CD133 and CD83, while IV20 cells were CD151 and CD38 (Supplementary Fig. 3a). Compared to late-response (IV1) cells, the early-response (IV20) cells exhibited an enhanced mesenchymal phenotype, as demonstrated by a decrease in the level of the epithelial marker E-cadherin (*CDH1*) and an increase in the levels of mesenchymal markers (e.g., Vimentin, Twist2, and Snail2) (Supplementary Fig. 3b).

### Inspection of the constructed BM-MSC-treated protein–protein interaction (PPI) network by systems biology approaches

**Fig. 2:**
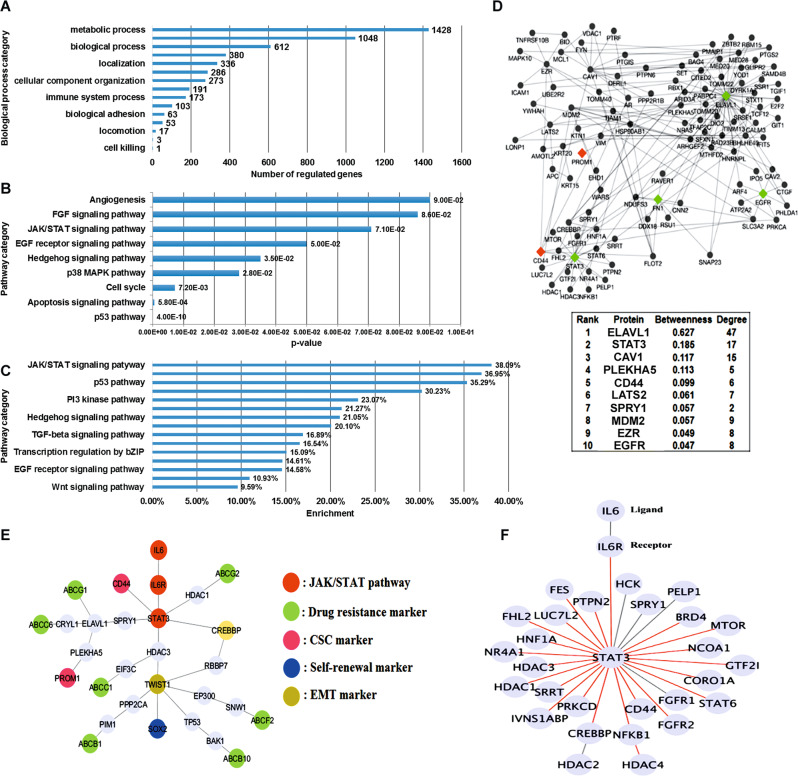
Inspection of the constructed PPI network in BM-MSC-treated cells by systems biology approaches. **A**–**C** Biological process analysis based on the constructed intracellular PPI network in BM-MSC-treated cells. The PPI network was constructed by PPI information from BioGRID and systems identification based on our proposed approach according to the microarray data at eight time points. The bar chart shows the numbers of regulated genes in the biological process categories as analysed using PANTHER. **D** The subnetwork extracted based on the cell surface markers. We extracted the subnetwork from the PPI network in BM-MSC-treated cells by expanding the lung cell surface markers CD44 and CD133 (also known as PROM1) until they connected with each other. The resulting subnetwork comprised 106 proteins and 183 interactions within the PPI network in BM-MSC-treated cells. **E** The subnetwork based on the proteins surrounding STAT3. The subnetwork was extracted from the PPI network in BM-MSC-treated cells to further investigate the role of STAT3 in the initialization and metastasis of lung CSCs after treatment with BM-MSC-secreted factors. STAT3 expression can potentially lead to the induction of drug resistance genes, cell surface markers, and EMT markers after treatment with BM-MSC-secreted factors. **F** The subnetwork based on the JAK/STAT pathway. A subnetwork with the possible connected interactions surrounding the IL6, IL6R, and STAT3 proteins was identified in the PPI networks in BM-MSC-treated cells. The red links indicate interactions that exist only in the subnetwork of the PPI network in BM-MSC-treated cells, and the gray links indicate interactions that appear in the PPI networks of both BM-MSC-treated and control cells.

Evidence is emerging that research in CSCs and systems biology could provide new insights into the design of novel therapeutic strategies for cancer patients. We apply a systems perspective into migrating cancer cells, and systems biology approaches would help us explore how PPIs and pathways are induced and controlled during early and late dissemination of lung cancer. Based on MB-MSCs-CM treated at eight time points microarray data and control microarray data, we constructed two intracellular PPI networks (the method of systems biology approaches as described in Supplementary information and Supplementary Fig. 4). By comparing these two networks, we could find out and predict the roles of the BM-MSCs-secreted factors in the initialization or metastasis of lung cancer. Our results showed that IL6/JAK/STAT [19–21] pathway might play a crucial role in this setting (Fig. 2A–D). We further identified STAT3 in our extracted subnetwork and crucial proteins, indicating that several CSC-related proteins, including drug resistance markers (ABCG1, ABCG2, ABCB1, ABCB10, ABCC1, ABCC6, and ABCF2), the stemness markers CD44 and CD133, the self-renewal marker SOX2, and the EMT marker Twist1, were closely related to JAK/STAT signaling (Fig. 2E). Moreover, many important genes were found to be connected to JAK/STAT signaling (Fig. 2F).

### BM-MSCs promote MET in HM20 CD133^+^/CD83^+^ early-response cells through activation of the LIFR/p-ERK/pS727-STAT3 signaling pathway

**Fig. 3:**
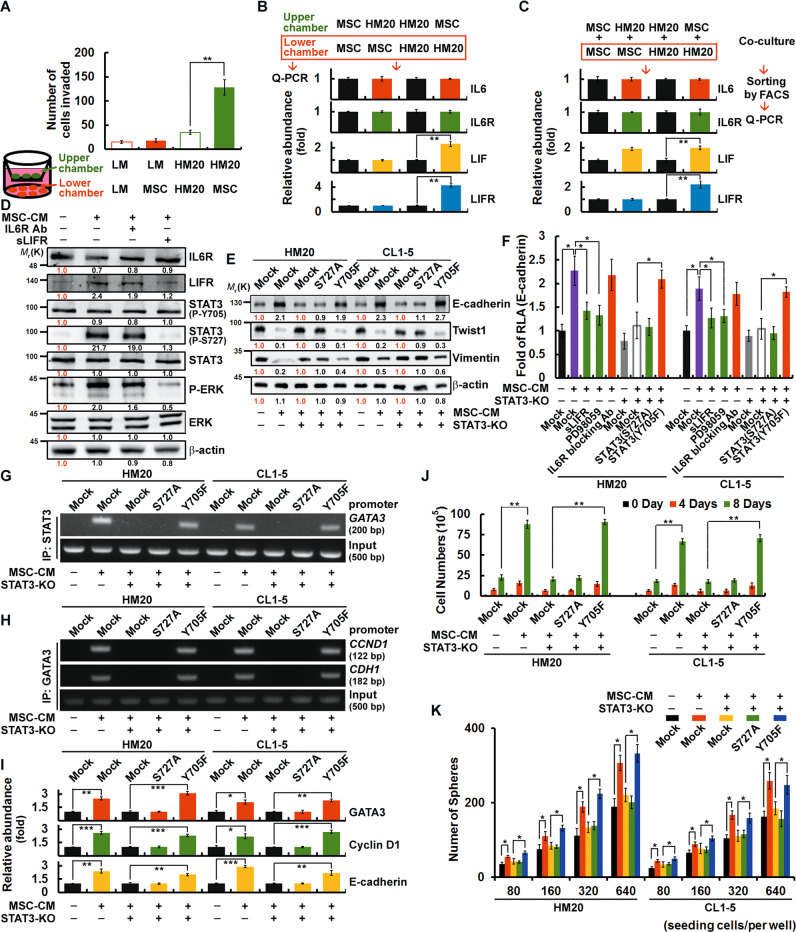
CD133^+^/CD83^+^ early-response (HM20) cells undergo STAT3/pS727-elicited MET to achieve metastatic colonization in a manner regulated by BM-MSCs. **A** Transwell invasion assays were performed to assess invasion. Cells were seeded in the upper and lower chambers as indicated. **B** Cells were seeded in the upper and lower chambers as indicated. The mRNA expression levels in the lower chambers were determined by QPCR. **C** Cells were co-cultured as indicated for 24 h. After sorting by FACS, the expression levels of the mRNAs in the lower panel were determined by QPCR. **D** HM20 cells were pretreated (for 24 h) with or without inhibitors (IL6R blocking antibody or soluble LIFR (sLIFR) to block the LIF/LIFR interaction) prior to incubation with BM-MSC-CM for 4 h. The expression levels of STAT3 signaling-related proteins were determined by western blotting. The relative intensities of the bands are shown. **E** STAT3 mutants were overexpressed in HM20 and CL1-5 STAT3-KO [an sgRNA was designed to target human *STAT3*, and STAT3 knockout was performed by CRISPR/Cas9 technology] cells prior to incubation with BM-MSC-CM for 24 h. The expression levels of EMT signaling-related proteins were determined by western blotting. The relative intensities of the bands are shown. **F** HM20 and CL1-5 STAT3-KO cells overexpressing STAT3 mutants were pretreated with inhibitors (for 24 h) prior to incubation with BM-MSC-CM for 24 h. A luciferase reporter assay was then performed to assess CDH1 promoter activity. **G**, **H** STAT3 mutants were overexpressed in HM20 and CL1-5 STAT3-KO cells prior to incubation with BM-MSC-CM for 24 h. For ChIP, DNA was immunoprecipitated with anti-STAT3 (in **G**) and anti-GATA3 (in **H**). The extracted DNA was analysed by PCR using primers spanning the proximal promoter regions of GATA3 (in **G**), CCND1 and CDH1 (in **H**). **I** The mRNA expression levels of cells described in (**G**) were determined by QPCR. **J** The total number of viable cells described in (**G**) was determined. **K** Cells described in (**G**) were cultivated in ultra-low-attachment 96-well plates under sphere-forming conditions. The numbers of spheres were calculated using microscopic analysis after 7 days. The data in (**A**–**C**), (**F**), and (**I**–**K**) were derived from three independent experiments and are presented as the mean values ± s.ds. *P < 0.05; **P < 0.01; ***P < 0.005 (*t*-test).

We first attempted to examine the mechanism by which BM-MSCs influenced HM20 early-response cells during the metastatic process. When HM20 cells were placed in the upper chamber and BM-MSCs in the lower chamber of a Transwell migration assay plate, the HM20 cells were attracted by and migrated towards the BM-MSCs (Fig. 3A). On the other hand, when BM-MSCs were placed in the upper chamber and HM20 cells in the lower chamber, increased expression of LIF and LIFR was observed in the HM20 cells (Fig. 3B). After these two types of cells were co-cultured, upregulation of LIF was observed in BM-MSCs, while increased expression of LIFR was seen in HM20 cells (Fig. 3C). BM-MSC-CM increased the expression of LIFR, p-ERK, and pS727-STAT3, and this activation was inhibited by a LIF inhibitor but not by an IL6R blocking antibody (Fig. 3D). These findings suggest that BM-MSCs secrete LIF and activate the LIFR/p-ERK/pS727-STAT3 signaling pathway in HM20 cells. Phenotypically, HM20 cells then transitioned from the mesenchymal state to a more epithelial-like state, as evidenced by a concomitant increase in the expression of E-cadherin and decrease in the expression of mesenchymal markers such as Twist1 and Snail1 (Fig. 3E). Furthermore, individually blocking each of the several components of the LIFR/p-ERK/pS727-STAT3 signaling pathway suppressed the transition of HM20 and CL1-5 cells to the epithelial phenotype, as measured by a reduction in their surface expression of E-cadherin (Fig. 3F). The chromatin immunoprecipitation (ChIP) assay results indicated that pS727-STAT3 was required for binding to the promoter of *GATA3* (Fig. 3G), which then bound to the promoter regions of cyclin D1 (*CCND1*) and E-cadherin (*CDH1*) (Fig. 3H). In summary, pS727-STAT3 was required for increasing the transcriptional activity of GATA3, cyclin D1, and E-cadherin (Fig. 3I). HM20 and CL1-5 cells were observed to exhibit enhanced proliferation (Fig. 3J) and sphere-forming abilities after BM-MSC-CM treatment (Fig. 3K). Here, we demonstrated that BM-MSCs activated the LIFR/p-ERK/pS727-STAT3 signaling pathway in HM20 and CL1-5 cells, which then underwent MET and acquired enhanced sphere-forming ability and proliferative potential. These two characteristics theoretically enable HM20 and CL1-5 cells to more effectively metastasize and colonize.

### BM-MSCs help LM CD151^+^/CD38^+^ late-response cells acquire CSC properties through the IL6R/pY705-STAT3 signaling pathway

**Fig. 4:**
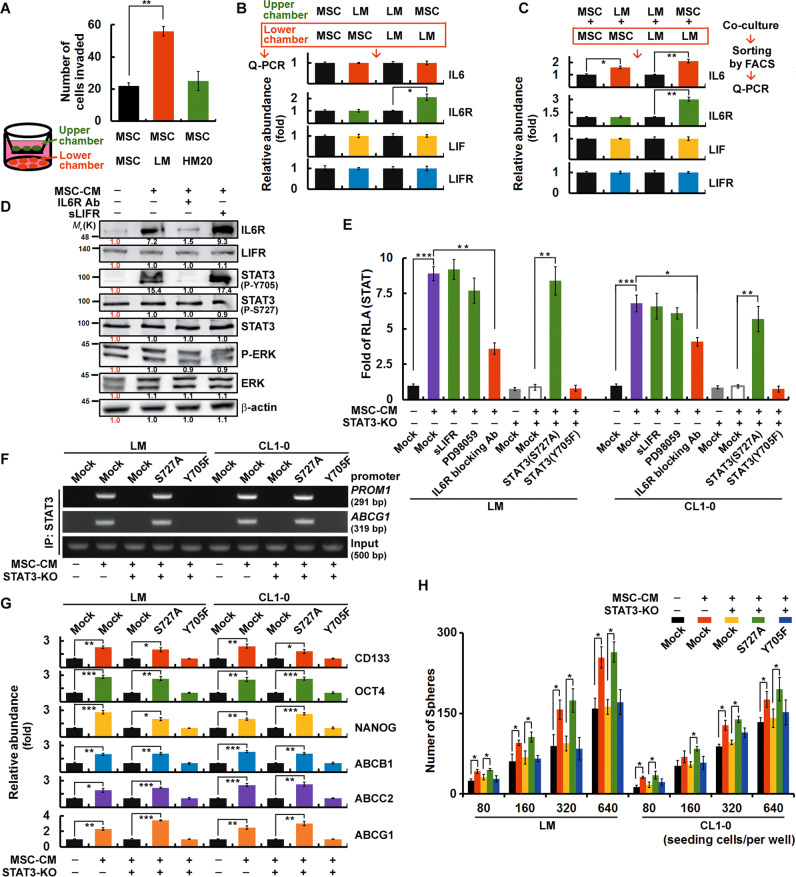
CD151^+^/CD38^+^ late-response (LM) cells undergo a STAT3/pY705-elicited gain of CSC properties in a manner regulated by BM-MSCs. **A** Transwell invasion assays were performed to assess invasion. Cells were seeded in the upper and lower chambers as indicated. **B** Cells were seeded in the upper and lower chambers as indicated. The mRNA expression levels in the lower chambers were determined by QPCR. **C** Cells were co-cultured as indicated for 24 h. After sorting by FACS, the expression levels of the mRNAs in the lower panel were determined by QPCR. **D** LM cells were pretreated (for 24 h) with or without inhibitors (IL6R blocking antibody or sLIFR to block the LIF/LIFR interaction) prior to incubation with BM-MSC-CM for 4 h. The expression levels of STAT3 signaling-related proteins were determined by western blotting. The relative intensities of the bands are shown. **E** LM and CL1-0 STAT3-KO [an sgRNA was designed to target human *STAT3*, and STAT3 knockout was performed by CRISPR/Cas9 technology] cells overexpressing STAT3 mutants or STAT3-Control cells (containing the nontargeting control sgRNA) were pretreated with inhibitors prior to incubation with BM-MSC-CM for 24 h. A luciferase reporter assay was then performed to assess STAT-responsive element activity. **F** STAT3 mutants were overexpressed in LM and CL1-0 STAT3-KO cells prior to incubation with BM-MSC-CM for 24 h. For ChIP, DNA was immunoprecipitated with anti-STAT3. The extracted DNA was analysed by PCR using primers spanning the proximal promoter regions of *PROM1* (CD133) and ABCG1. **G** The mRNA expression levels of cells described in (**F**) were determined by QPCR. **H** Cells described in (**F**) were cultivated in ultra-low-attachment 96-well plates under sphere-forming conditions. The numbers of spheres were calculated using microscopic analysis after 7 days. The data in (**A**–**C**), (**E**), and (**G**–**H**) were derived from three independent experiments and are presented as the mean values ± s.ds. *P < 0.05; **P < 0.01; ***P < 0.005 (*t*-test).

When BM-MSCs were placed in the upper chamber of the Transwell invasion assay plate, a significantly higher number of BM-MSCs was observed to migrate towards the LM cells in the lower chamber than towards the control cells (BM-MSCs) or HM20 cells (Fig. 4A). The soluble factors released by the BM-MSCs in the upper chamber increased the expression of IL6R at both the RNA and protein levels in the LM cells in the lower chamber, as demonstrated by QPCR and western blotting (Fig. 4B–D). QPCR of FACS-sorted co-cultured BM-MSCs and LM cells showed increased expression of IL-6 in BM-MSCs and upregulation of both IL6 and IL6R in LM cells (Fig. 4C). BM-MSC-CM activated the IL6R/pY705-STAT3 signaling pathway, and this effect was inhibited by the IL6R blocking antibody but not by the LIFR inhibitor (Fig. 4D). On the transcriptional level, the luciferase reporter assay results also indicated that BM-MSC-CM activated STAT3 transcriptional activity via the IL6R/pY705-STAT3 pathway in LM and CL1-0 cells (Fig. 4E). Furthermore, the ChIP assay results indicated that pY705-STAT3 was required for the binding of STAT3 to the promoters of CD133 (*PROM1*) and *ABCG1* (Fig. 4F), as well as for upregulating the expression of several CSC-related genes, such as CD133, Oct4, Nanog, ABCA3, ABCB1, ABCC2, and ABCG1 (Fig. 4G). BM-MSC-CM also enhanced the sphere-forming ability of LM and CL1-0 cells (Fig. 4H). In summary, BM-MSCs migrated towards LM and CL1-0 cells, subsequently activating the IL6R/pY705-STAT3 signaling pathway and ultimately leading to the acquisition of CSC characteristics by LM and CL1-0 cells.

### BM-MSCs elicit EMT in LM CD151^+^/CD38^+^ late-response cells through the IL6R/pY705-STAT3 signaling pathway

**Fig. 5:**
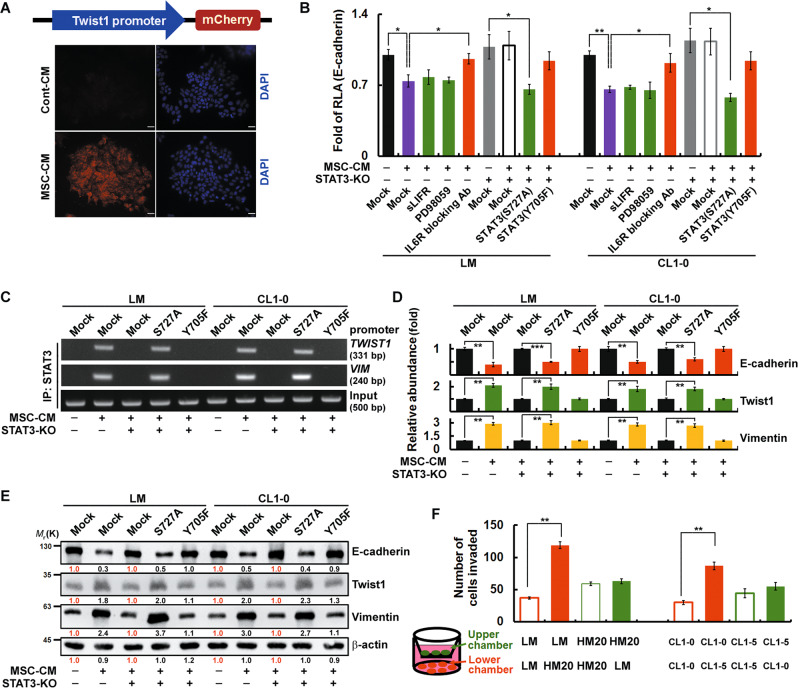
CD151^+^/CD38^+^ late-response (LM) cells undergo STAT3/pY705-elicited EMT to achieve tumor heterogeneity and cancer cell plasticity at the metastatic site in a manner regulated by BM-MSCs. **A** LM cells were treated with BM-MSC-CM. A fluorescent (mCherry) reporter assay was then performed to assess Twist1 promoter activity. **B** LM and CL1-0 STAT3-KO cells overexpressing STAT3 mutants or STAT3-Control cells were pretreated (for 24 h) with inhibitors prior to incubation with BM-MSC-CM for 24 h. A luciferase reporter assay was then performed to assess *CDH1* (E-cadherin) promoter activity. **C** STAT3 mutants were overexpressed in LM and CL1-0 STAT3-KO cells prior to incubation with BM-MSC-CM for 24 h. For ChIP, DNA was immunoprecipitated with anti-STAT3. The extracted DNA was analysed by PCR using primers spanning the proximal promoter regions of Twist1 and VIM. **D** The mRNA expression levels of cells described in (**C**) were determined by QPCR. **E** The expression levels of EMT signaling-related proteins were determined by western blotting. The relative intensities of the bands are shown. **F** Transwell invasion assays were performed to assess invasion. After BM-MSC-CM treatment, cells were seeded in the upper and lower chambers as indicated. The data in (**B**), (**D**), and (**F**) were derived from three independent experiments and are presented as the mean values ± s.ds. *P < 0.05; **P < 0.01; ***P < 0.005 (*t*-test).

In the reporter assay, LM cells exhibited elevated Twist1 expression after BM-MSC-CM treatment (Fig. 5A). BM-MSC-CM treatment decreased the transcriptional activity of E-cadherin in LM and CL1-0 cells, while addition of the IL6R blocking antibody reversed this repression (Fig. 5B). In addition, BM-MSC-CM treatment did not reduce the transcriptional activity of E-cadherin in cells expressing the non-phosphorylatable STAT3^Y705F^ mutant. The ChIP assay results confirmed that pY705-STAT3 was required for the binding of STAT3 to the *Twist1* promoter (Fig. 5C). Subsequently, the expression of mesenchymal markers such as Twist1 and vimentin was enhanced, and the expression of E-cadherin was suppressed (Fig. 5D, E). Taken together, these findings indicate that BM-MSCs direct LM and CL1-0 cells to undergo EMT through IL6R/STAT3-Y705 signaling. Notably, LM and CL1-0 cells were attracted by and migrated towards HM20 and CL1-5 cells respectively (Fig. 5F), which, theoretically, were more abundant than other cell types in the secondary site.

### Elevated expression of LIFR and phosphorylation of S727-STAT3 are correlated with disease progression and distant metastasis in patients with lung cancer

**Fig. 6:**
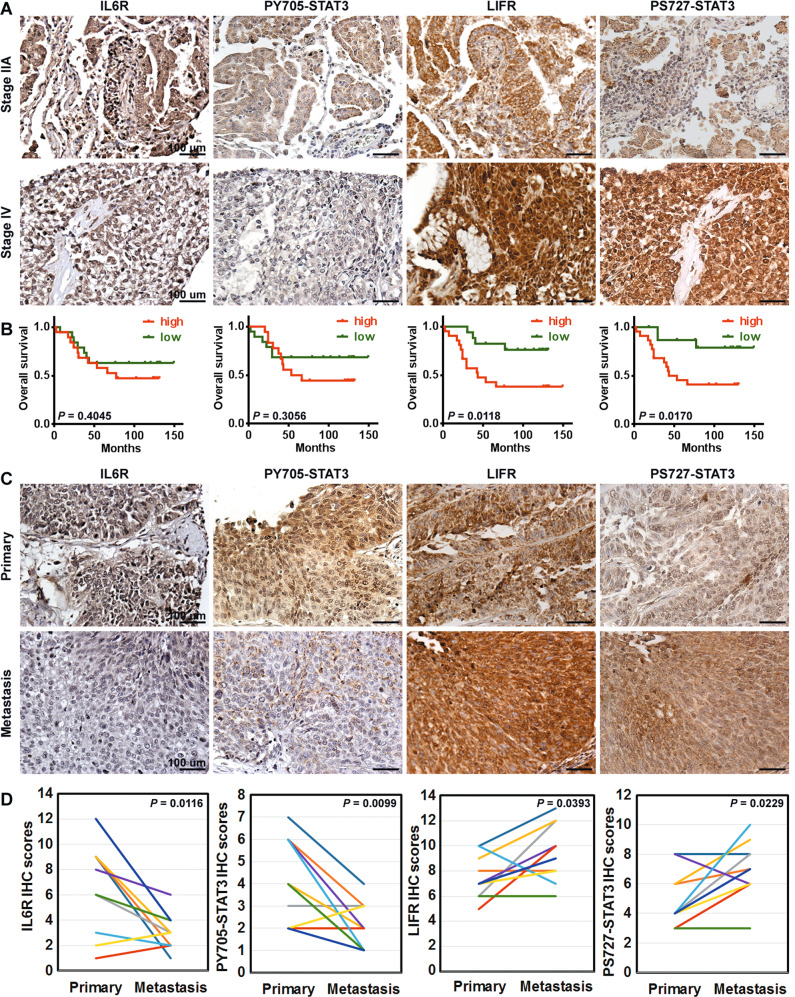
Elevated expression of LIFR and phosphorylation of S727-STAT3 are clinically significant. Clinical significance of IL6R, pY705-STAT3, LIFR, and pS727-STAT3 in patients with lung cancer. **A** IHC analysis of lung cancer tissue array samples for IL6R, pY705-STAT3, LIFR, and pS727-STAT3. Both low-grade (stage IIA) and high-grade (stage IV) cancer specimens are shown. Staging of the primary cancers was carried out according to the AJCC Cancer Staging Manual (7th edition). Tissues were counterstained with haematoxylin. Bars, 100 µm. **B** Kaplan–Meier estimation of overall survival stratified by the expression levels of IL6R, pY705-STAT3, LIFR, and pS727-STAT3. Via a bimodal IHC score distribution, tissues with IHC scores lower than the average score were designated as having low expression, and those with scores higher than the average score were designated as having high expression. **C**, **D** Changes in the levels of IL6R, pY705-STAT3, LIFR, and pS727-STAT3 between primary and metastatic samples (bone).

We then examined whether cancer in different stages is preferentially related to activation of either the IL6R/pY705-STAT3 or the LIFR/pS727-STAT3 pathway. The expression profiles of IL6R, pY705-STAT3, LIFR, and pS727-STAT3 were determined based on immunohistochemical (IHC) staining and scoring of consecutive slides from 40 primary (stages IA, IB, IIA, IIB, III, and IV) human lung cancer specimens. High-grade (stage IV) primary lung cancers showed higher levels of LIFR and pS727-STAT3 than low-grade (stage IIA) cancers (Fig. 6A). As expected, univariate analysis indicated that an expression profile of LIFR^high^ or pS727-STAT3^High^ was correlated with shorter overall survival times (Fig. 6B). Therefore, identifying a LIFR^high^/pS727-STAT3^High^ molecular profile is a potential strategy for monitoring disease progression in lung cancer. Furthermore, differential phosphorylation of STAT3 was observed between primary tumors and their metastatic counterparts from ten patients. Primary tumors were found to have higher expression levels of IL6R and pY705-STAT3, while metastatic tumors had higher expression levels of LIFR and pS727-STAT3 (Fig. 6C, D).

### Heterogeneous CSC subtypes undergo STAT3/pY705-elicited EMT and STAT3/pS727-elicited MET to achieve metastatic colonization in a manner regulated by MSCs in vivo

For the in vivo bone homing assay, mice were sacrificed on day 5 after intracardiac inoculation; then, the hind limbs were flushed, and cells were isolated and cultured. Single-cell–derived colonies (SCDCs) were counted under a light microscope after crystal violet staining. Consistent with previous results, the number of SCDCs was dramatically increased in the hind limbs of animals after co-culture with MSCs under sphere-forming conditions (Supplementary Fig. 5). These effects were abolished by either the STAT3^S727A^ mutation in early-response (HM20) cells or the STAT3^Y705F^ mutation in late-response (LM) cells (Supplementary Fig. 5).

**Fig. 7:**
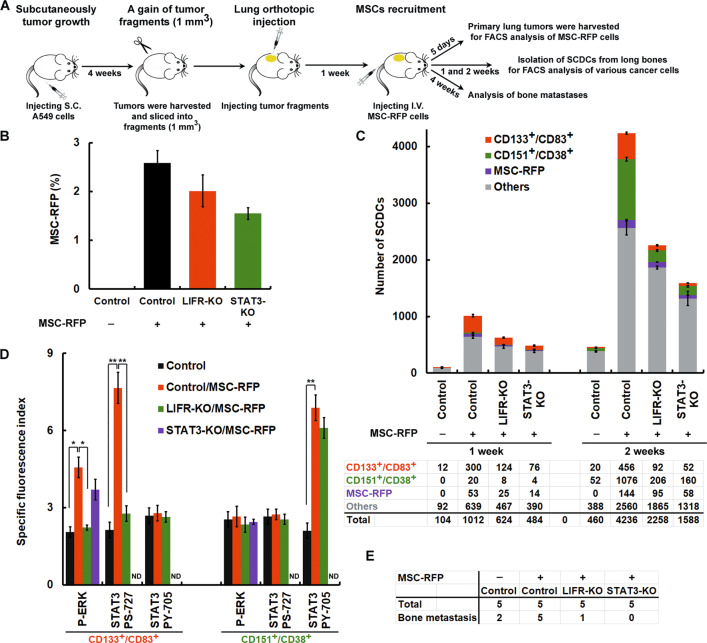
Heterogeneous CSC subtypes undergo STAT3/pY705-elicited EMT and STAT3/pS727-elicited MET to achieve metastatic colonization in a manner regulated by MSCs in vivo. **A** Diagram depicting the procedure to test whether MSC-elicited STAT3 signaling involved in lung cancer bone metastases. The details are summarized in Supplementary Materials and methods. **B** MSC recruitment assay. After lung orthotopic injection for 1 week, 0.5 × 10^6^ MSC-RFP cells were injected intravenously. The primary lung tumors were harvested after 5 days for FACS analysis of MSC-RFP cells. Percent MSC-RFP cells in the primary tumor was shown. **C**–**E** Analysis of bone metastases. After MSC-RFP cells injection for 1 and 2 weeks, SCDCs were isolated from long bones after for FACS analysis of various cancer cells. The number of bone metastatic cells was shown in (**C**). Bone metastatic (CD133^+^/CD83^+^ and CD151^+^/CD38^+^) cells were incubated with isotype IgG (control) or antibody specifically recognizing p-ERK, pY705-STAT3, and pS727-STAT3 followed by labeling with Alexa Fluor 488-conjugated secondary antibody. Fluorescence intensity was determined with flow cytometry. The specific fluorescence index (SFI) was calculated as the ratio of the geometric mean fluorescence value obtained with the specific antibody and the isotype control antibody (shown in **D**). The performance status of the mice began to decrease, at which time the animals were sacrificed and autopsied. The orthotopic primary tumor and all major organs as well as the whole skeleton were explored. The incidence of bone metastases was shown in (**E**). ND not detectable. *P < 0.05; **P < 0.01.

Furthermore, we established an experimental model of bone metastasis by human lung cancer cells (Fig. 7A). For subcutaneously tumor growth, mice were injected subcutaneously with A549 cells. Tumorigenicity was evaluated at 4 weeks after transplantation. For lung orthotopic injection [22], tumor fragments (1 mm^3^) derived from the A549 subcutaneously tumor were implanted by lung orthotopic injection. For MSC recruitment assay [23], 0.5 × 10^6^ MSC-RFP cells were injected intravenously after lung orthotopic injection for 1 week. The primary lung tumors were harvested after 5 days for FACS analysis of MSC-RFP cells. In cell suspensions prepared from harvested tumors, we found that MSC-RFP cells were located in the primary lung tumors and percent MSC-RFP cells was 1.5–2.5% (Fig. 7B). For analysis of bone metastases. SCDCs were isolated from long bones after for FACS analysis of various cancer cells after MSC-RFP cells injection for 1 and 2 weeks. After MSC-RFP cells injection for 1 week, most of bone metastatic cells were CD133^+^/CD83^+^ (early-response) cells (Fig. 7C, left panels) and the LIFR/p-ERK/pS727-STAT3 signaling pathway was activated (Fig. 7D, left panels). The effects can be abolished by LIFR and STAT3-knockout in A549 cells. After MSC-RFP cells injection for 2 weeks, CD151^+^/CD38^+^ (late-response) cells were dramatically increased in bone metastatic cells (Fig. 7C, right panels) and pY705-STAT3 was enhanced (Fig. 7D, right panels). The effects only can be abolished by STAT3-knockout in A549 cells. The performance status of the mice began to decrease, at which time the animals were sacrificed and autopsied. After MSC-RFP cells injection for 4 weeks, bone metastases were all found in 5 mice. LIFR and STAT3-knockout in A549 cells can decrease bone metastases (Fig. 7E). Taken together, we suggest that STAT3/pY705-elicited EMT and STAT3/pS727-elicited MET are required for the achievement of metastatic colonization in a manner regulated by MSCs in vivo.

**Fig. 8:**
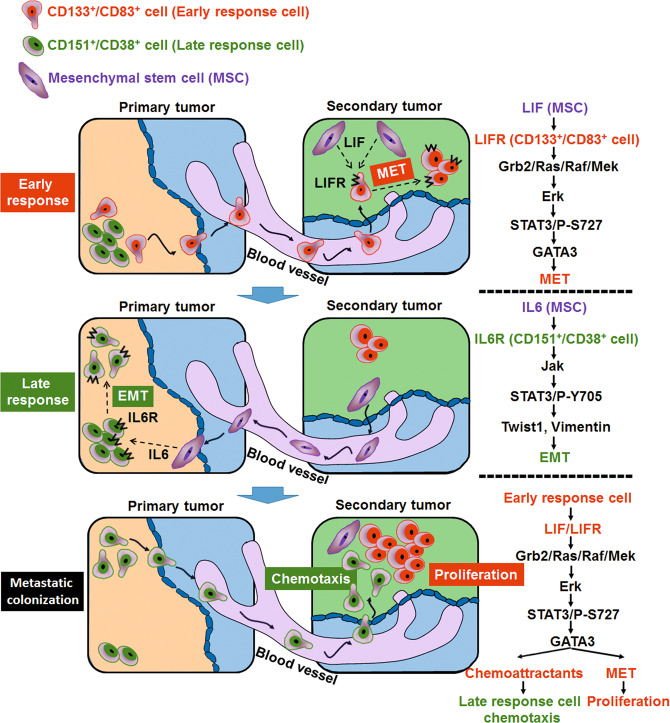
A model for STAT3 phosphorylation at Ser727 and Tyr705 differentially regulating the EMT–MET switch and cancer metastasis. Model proposing a pathway in which heterogeneous CD151^+^/CD38^+^ and CD133^+^/CD83^+^ CSC subtypes undergo STAT3/pY705-elicited EMT and STAT3/pS727-elicited MET, respectively, to achieve metastatic colonization in a manner regulated by MSCs.

Here, we constructed a model describing the mechanism by which BM-MSCs introduce heterogenicity into the CSC niche, promoting tumor metastasis and secondary site homing (Fig. 8). In primary tumors, the subgroup of cells that responds early to BM-MSC-CM is termed HM20 (CD133^+^/CD83^+^) cells. Upon BM-MSC signaling, HM20 cells migrate towards the secondary site, where BM-MSCs are abundant. BM-MSC-induced upregulation of LIF and LIFR in HM20 cells activates ERK, which enhances STAT3 phosphorylation at S727. Through this pathway, HM20 cells proliferate and undergo MET while still maintaining their CSC properties. These characteristics enable efficient metastatic colonization. After the first subgroup of cells migrates to the secondary site, BM-MSCs circulate to the primary site, where the second subgroup of cells, which respond slowly to BM-MSC-CM, are located. The IL6R level in these cells, called LM cells, increases upon BM-MSC stimulation. This increase activates the JAK/STAT3 pathway, which leads to an increase in STAT3 phosphorylation at Y705. As their CSC properties are enhanced, LM cells then undergo EMT and migrate towards the secondary site, where HM20 cells have colonized. This 2-step metastasis process carried out by two heterogeneous subgroups of cancer cells is then completed.

## Discussion

This work provides evidence that human lung cancer cells migrate and disseminate from morphologically very early lesions. The mechanism consists of three major components: the niche, the EMT–MET program and STAT3 signaling. While the specific molecular details are more likely to be tissue dependent than universal, our proposed mechanism may provide a general framework for the understanding of metastasis with cancer cells undergoing a switch from a dissemination to proliferation mode. We observed that cells with a CD133^+^/CD83^+^ signature exhibited early migratory ability (early-response cells) and a mesenchymal phenotype. We believe that these early-response HM20 cells were analogous to eDCCs. These cells migrated towards BM-MSCs in the invasion assay and, upon treatment with BM-MSC-CM, exhibited enhanced LIFR/p-ERK/pS727-STAT3 signaling, which resulted in increased transcriptional activity of GATA3, cyclin D1, and E-cadherin. These early-response HM20 cells also demonstrated an ability to proliferate and form spheres in suspension culture. Our findings also demonstrated the concept that late-disseminating, fully mature cancer cells necessarily have a higher ability to form metastases. After treatment with BM-MSC-CM, these late responders had a CD151^+^/CD38^+^ molecular signature and were characterized by their relatively low motility (LM cells) as well as their increased expression of epithelial markers and reduced expression of mesenchymal markers (Fig. 1F). In the invasion assay, BM-MSCs migrated towards these CD151^+^/CD38^+^ LM cells in the lower chamber (Fig. 4A), a finding that lends support to previous evidence of BM-MSC tropism for tumors [15]. Upon continued treatment with BM-MSC-CM, CD151^+^/CD38^+^ LM cells exhibited activated IL-6 receptor/pY705-STAT3 signaling, which led to upregulation of Twist1 and downregulation of E-cadherin expression (Fig. 5D, E). Based on these findings, we consider these CD151^+^/CD38^+^ LM cells to be a model for the cells in the primary tumor that are capable of metastasis.

Transient de-differentiation (EMT)–re-differentiation (MET) processes are proposed to be a driving force of cancer metastasis; however, considerable uncertainty regarding the EMT–MET switch remains: (1) Although many clinical reports [24] have advanced the concept of transient EMT–MET switches in metastasis, there are only a few experimental proofs. (2) EMT is proposed to provide cancer cells with several prometastatic traits, including motility and stemness. One argument that has been raised against a role for EMT in cancer progression is that metastatic tumors examined histologically often exhibit an epithelial-like phenotype and resemble the primary tumor. (3) Many studies support the need for re-differentiation (MET) to support the colonization and metastasis of differentiated carcinomas and show that EMT-associated growth arrest is a reason underlying this need. However, how do metastases re-differentiate? (4) Future therapeutic strategies against metastasis will have clinical impacts. Inducing differentiation and targeting EMT alone might be counterproductive by inducing the proliferation of disseminated cells. How should this problem be solved? Furthermore, CSCs have been shown to exhibit different phenotypes depending on their intratumoral location, with the cells at the invasive front demonstrating a mesenchymal state and the cells in the central part exhibiting an epithelial state [25]. Biddle et al. found that the mesenchymal phenotype is preferentially migratory and the epithelial phenotype proliferative [26]. Both the HM20 CD133^+^/CD83^+^ cells and the LM CD151^+^/CD38^+^ cells in our study, despite their phenotypic differences, formed spheres in suspension culture, a feature characteristic of cellular stemness. Our findings correspond well with Brabletz’s concept of “migrating CSCs” [27], which stipulates that malignant stem cells need to acquire one phenotype associated with growth and another that is migratory and characterized by "transient expression of EMT-associated genes, which can be reversed by MET, leading to epithelial re-differentiation" in order to form metastatic colonies at a distant site.

LIF is a pleiotropic member of the IL-6 family of cytokines. LIF mediates the JAK/STAT3, PI3K, and ERK1/2 signaling pathways and regulates cell proliferation, differentiation, and survival [28]. The mechanism by which LIF participates in tumorigenesis, however, remains largely unexplored. In practice, LIF is used to maintain embryonic stem cells in a totipotent state and stimulate self-renewal [29], as it activates the JAK/pY705-STAT3 pathway and, subsequently, the expression of pluripotency genes [30]. A recent study by Huang et al. demonstrated that different culture environments resulted in differential phosphorylation of STAT3 in embryonic stem cells, with LIF-STAT3 pS727 signaling directing the cells towards neuronal differentiation [31]. The results of that study suggest that STAT3 can be differentially activated to regulate cell differentiation and phenotypic determination. Our data show that BM-MSCs elicit EMT in epithelial-type cells through the IL-6/pY705-STAT3 pathway and induce MET in mesenchymal-type cells through LIFR/pS727-STAT3 signaling. These findings support the idea that stem or stem-like cells may use differential STAT3 phosphorylation as a means to control their fate.

In conclusion, our data suggest that BM-MSCs elicit MET in HM20 metastatic cancer cells through LIFR/pS727-STAT3 signaling, potentially resulting in cancer cell proliferation and the establishment of macroscopic colonies. On the other hand, BM-MSCs aid in EMT of LM tumor cells in the primary site through IL-6-induced phosphorylation of the Y705 residue on the STAT3 molecule. These results reaffirm the concept of tumor heterogeneity and imply that different subsets of cells within a tumor can respond differently to the same stimulus based on their specific cellular context and the local niche that they reside in.

## Materials and methods

### Cell lines

All cell lines were obtained from the ATCC. They were tested and authenticated by short tandem repeat analysis and tested for mycoplasma contamination.

### CRISPR/Cas9 genome editing

For STAT3-knockout experiments, the pAll-Cas9.pPuro lentiviral vector containing the single-guide RNA (sgRNA) targeting human STAT3 and the lentiviral vector containing the nontargeting control sgRNA were purchased from the National RNAi Core Facility, Academia Sinica, Taipei, Taiwan [32]. The sequence of the STAT3-targeting sgRNA was GCAGCTTGACACACGGTACC. Cells were transfected with plasmids carrying the individual sgRNAs. Homozygous STAT3 knockout cells were selected by serial dilution and single-cell culture. The knockout efficiency was confirmed by western blotting.

### Constructs and reagents

Wild-type STAT3 was purchased from Genediscovery Biotechnology. The tyrosine mutants (STAT3^Y705F^ and STAT3^S727A^) were constructed by site-directed mutagenesis using the wild-type STAT3 cDNA template [33]. The correct sequence of the clones was verified by sequencing. Antibodies against the following proteins were used: CD38 (555459), CD44 (555478), CD45 (555485), and E-cadherin (610181) (all from BD Biosciences); CD105 (130-098-774) and CD133 (130-080-801) (both from Miltenyi Biotec); CD83 (11-0839-42; eBioscience); Vimentin (IF01; Calbiochem); CD151 (FAB1884P; R&D Systems); IL6R (sc-373708) and STAT3 (sc-8019) (both from Santa Cruz Biotechnology); GATA3 (ab199428), LIFR (ab202847), and Twist1 (Ab50887) (all from Abcam); β-actin (A5441), β-catenin (C2206) and TCF4 (T5817) (all from Sigma); and ERK (9102S), Snail1 (3895S), phospho-ERK (9101S; phosphorylated at T202/Y204), phospho-STAT3 (9138; phosphorylated at Y705) and phospho-STAT3 (9136; phosphorylated at S727) (all from Cell Signaling Technology).

### Statistical analysis

Statistical analysis of data was performed with Student’s *t*-test using SigmaPlot software. The Spearman correlation coefficient was used to assess the association between two continuous variables. Differences were statistically significant at *P* < 0.05.

## Supplementary information

Supplementary Information
