# Supplementary material for: STAT3 phosphorylation at Ser727 and Tyr705 differentially regulates the EMT–MET switch and cancer metastasis
Source: Oncogene. 2020 Dec 1;40(4):791–805. doi: 10.1038/s41388-020-01566-8 (PMC7843420; doi:10.1038/s41388-020-01566-8)
Supplement: Supplementary file 1 — Supplementary Information [file 41388_2020_1566_MOESM1_ESM.pdf]

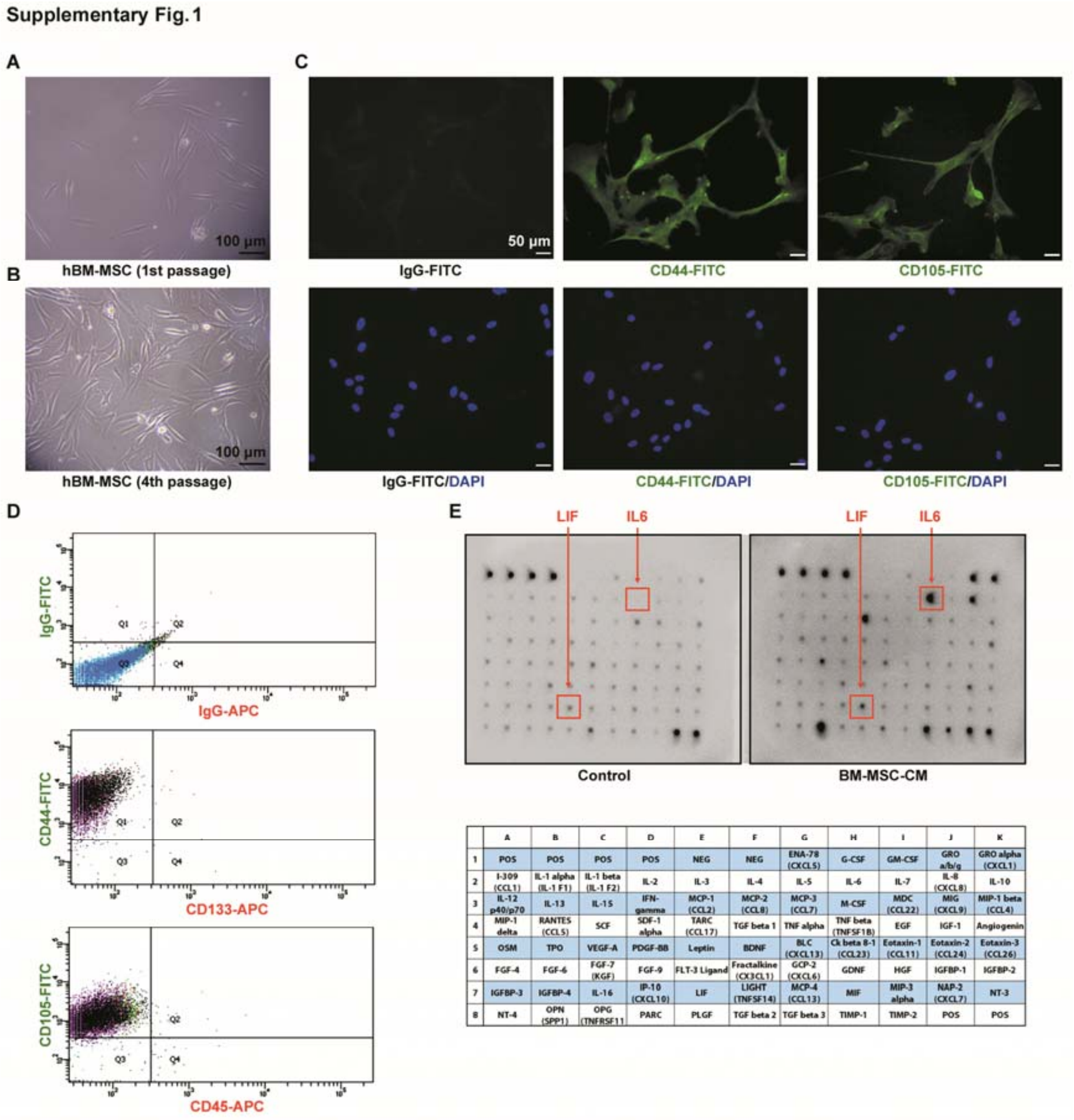

	A	B	C	D	E	F	G	H	I	J	K
1	POS	POS	POS	POS	NEG	NEG	ENA-78 (CXCL5)	G-CSF	GM-CSF	GRO a/b/g	GRO alpha (CXCL1)
2	I-309 (CCL1)	IL-1 alpha (IL-1 F1)	IL-1 beta (IL-1 F2)	IL-2	IL-3	IL-4	IL-5	IL-6	IL-7	IL-8 (CXCL8)	IL-10
3	IL-12 p40/p70	IL-13	IL-15	IFN-gamma	MCP-1 (CCL2)	MCP-2 (CCL8)	MCP-3 (CCL7)	M-CSF	MDC (CCL22)	MIG (CXCL9)	MIP-1 beta (CCL4)
4	MIP-1 delta	RANTES (CCL5)	SCF	SDF-1 alpha	TARC (CCL17)	TGF beta 1	TNF alpha	TNF beta (TNFSF1B)	EGF	IGF-1	Angiogenin
5	OSM	TPO	VEGF-A	PDGF-BB	Leptin	BDNF	BLC (CXCL13)	Ck beta 8-1 (CCL23)	Eotaxin-1 (CCL11)	Eotaxin-2 (CCL24)	Eotaxin-3 (CCL26)
6	FGF-4	FGF-6	FGF-7 (KGF)	FGF-9	FLT-3 Ligand	Fractalkine (CX3CL1)	GCP-2 (CXCL6)	GDNF	HGF	IGFBP-1	IGFBP-2
7	IGFBP-3	IGFBP-4	IL-16	IP-10 (CXCL10)	LIF	LIGHT (TNFSF14)	MCP-4 (CCL13)	MIF	MIP-3 alpha	NAP-2 (CXCL7)	NT-3
8	NT-4	OPN (SPP1)	OPG (TNFRSF11	PARC	PLGF	TGF beta 2	TGF beta 3	TIMP-1	TIMP-2	POS	POS

Supplementary Figure 1. Isolation and characterization of human BM-MSCs

a, b Morphology of human BM-MSCs at the 1st passage and the 4th passage. Scale bars = 100 µm. **c** Immunostaining for the MSC-positive markers CD44 and CD105 is shown in isolated cells. Scale bars = 50 µm. **d** Flow cytometric analysis of the surface markers CD44, CD45, CD105 and CD133 in isolated human BM-MSCs. The isolated human BM-MSCs were positive for CD44 and CD105 and negative for CD133 and CD45. **e** A human cytokine antibody array (the human Cytokine Array C5, AAH-CYT-5-4, RayBiotech) was used to measure the secretion of cytokines in control medium (Control) and human BM-MSC-CM to represent tumor-conditioned medium. The

factors that were more highly produced in human BM-MSC-CM than in control medium are shown within the rectangular outlines.

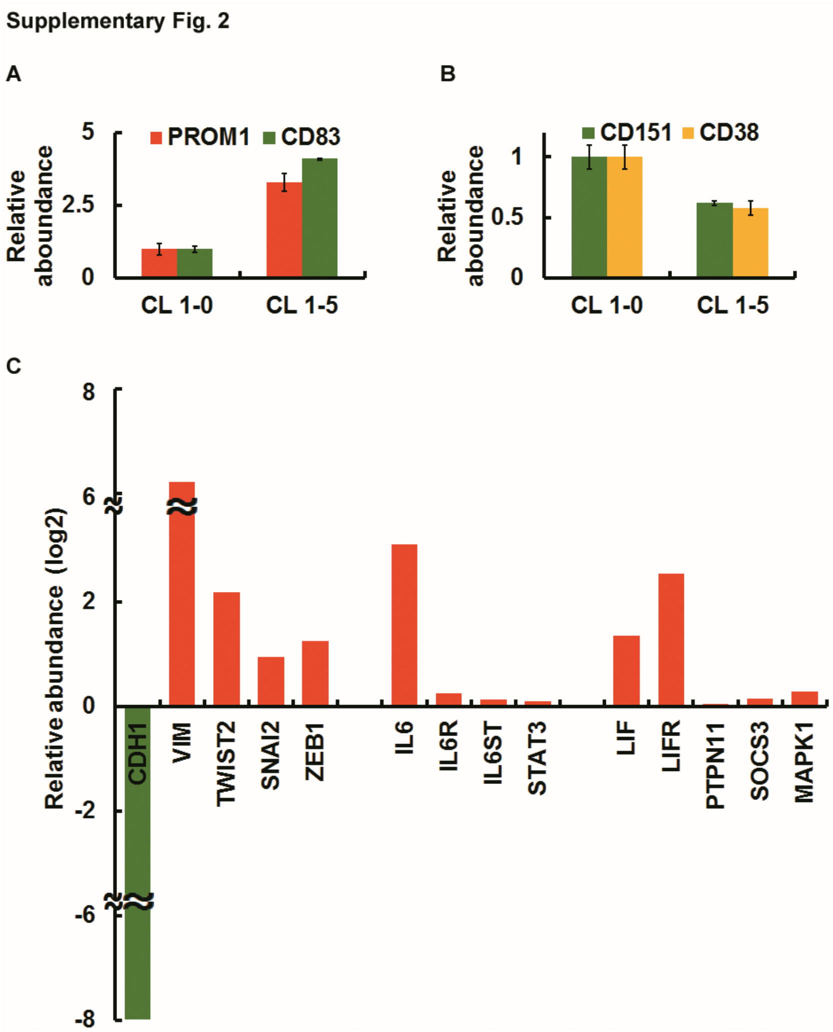

Supplementary Figure 2. Identification of a gene expression signature linked to early dissemination using CL1-0 and CL1-5 cells

a, b The mRNA expression levels of surface markers were determined by QPCR [CD133 (*PROM1*) and CD83 in a; CD151 and CD38 in b]. **c** QPCR showing the mRNA expression levels of STAT3 signaling-related genes. CL1-5 cells are compared to CL1-0 cells. The data in (a) and (b) were derived from three independent experiments and are presented as the mean values±s.ds.

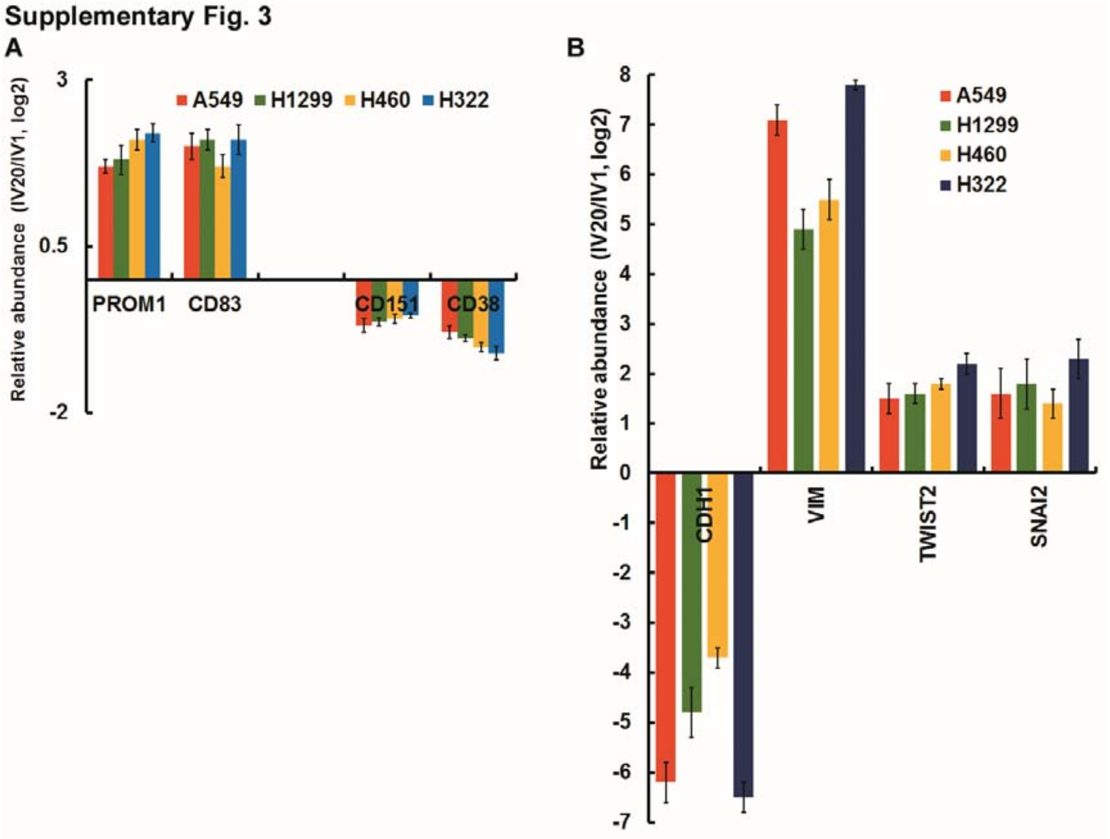

Supplementary Figure 3. Identification of a gene expression signature linked to early dissemination using A549, H1299, H460 and H322 lung cancer cells

Functional fractionation was performed on A549, H1299, H460 and H322 lung cancer cells by subjecting them to an invasion assay. The cells that remained in situ were designated IV1 cells. The IV1 cell phenotype was further enhanced and stabilized through 19 rounds of invasion assays (under BM-MSC-CM stimulation), with the invaded cells harvested from each subsequent round of selection designated IV2 to IV20 cells. **a** The mRNA expression levels of surface markers were determined by QPCR [CD133 (*PROM1*), CD83, CD151, and CD38]. IV20 cells are compared to IV1 cells. **b** QPCR showing the mRNA expression levels of EMT-related genes. IV20 cells are compared to IV1 cells. The data in (a) and (b) were derived from three independent experiments and are presented as the mean values±s.ds.

Supplementary Fig. 4

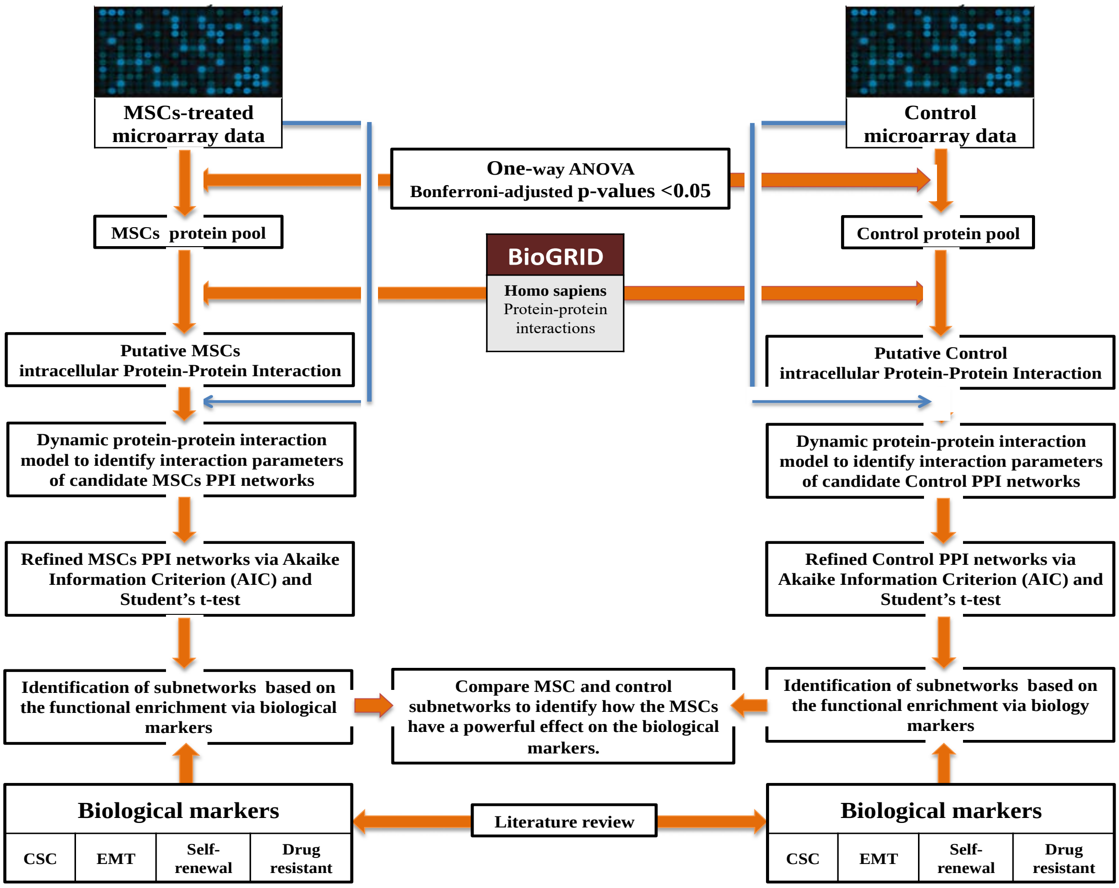

Supplementary Figure 4. Flow chart describing the construction of the MSCs-treated and Control intracellular PPI network

The figure shows the flowchart of the proposed approaches. One-way ANOVA was applied to select significant protein into protein pools. PPIs of Homo sapiens data were obtained from BioGRID database, and then we obtained the putative intracellular MSCs and control PPIs by protein pools and BioGRID database. Moreover, the candidate PPI network was constructed by using a dynamic PPI model. By Akaike information criterion (AIC) and Student's t-test, MSCs and control candidate PPI networks were refined by system order selection and by determining significance of the protein interactions. Finally, comparing sub-networks extracted from the construction of the

MSCs-treated and Control networks via CSCs markers, we investigate the influence of MSCs-secreted factors on each of CSCs markers.

Supplementary Fig. 5

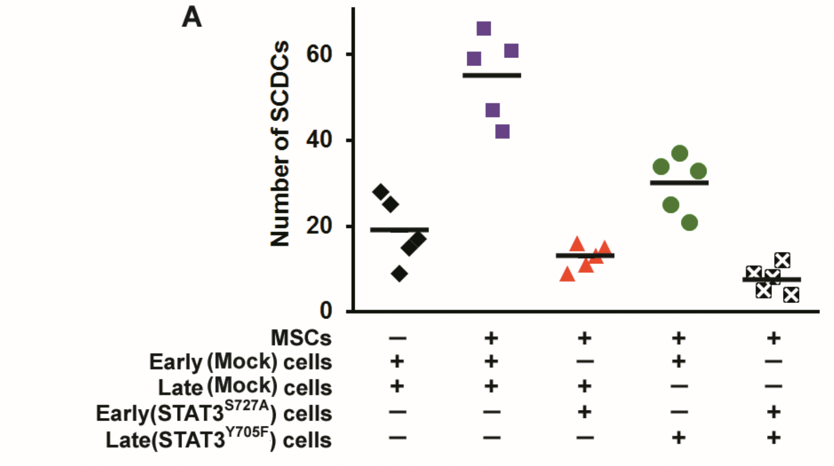

Supplementary Figure 5. The *in vivo* bone homing assay

STAT3 mutants were overexpressed in LM/STAT3-knockout and HM20/STAT3-knockout cells prior to co-culture with MSCs in suspension. For the bone homing assay, 2×10^5 (1×10^5 LM and 1×10^5 HM20 cells) or 3×10^5 (the above cells co-cultured with 1×10^5 MSCs) cells were cultured under sphere-forming conditions for 7 days before injection (a volume of exactly 100 µL) into the left cardiac ventricle of 6-week-old mice. Mice were sacrificed on day 5 after intracardiac inoculation. The hind limbs were flushed, and cells were isolated and cultured. SCDCs were counted under a light microscope after crystal violet staining.

Supplementary materials and methods

Isolation and characterization of human BM-MSCs

Fresh human BM was obtained from healthy volunteers during surgery. This study was approved by the institutional review board (IRB) of National Taiwan University Hospital Hsin-Chu Branch. Density gradient centrifugation was performed to isolate mononuclear cells from human BM. Mononuclear cells were isolated from BM aspirates by density gradient centrifugation (Ficoll 1.077 g/mL) and plated in non-coated 75- to 175-cm^2 polystyrene culture flasks (Corning Costar) at a density of 160,000 cells/cm^2 in complete culture medium: Mesencult (Stem Cell Technologies) supplemented with 10% foetal calf serum (FCS; MSC Stimulatory Supplements, Stem Cell Technologies), 2 mmol/L L-glutamine, and 50 μg/mL gentamicin (Life Technologies). Cultures were maintained at 37°C in a humidified atmosphere containing 5% CO_2. After 48 hours of adhesion, non-adherent cells were removed, and the culture medium was replaced twice weekly. BM-MSCs were harvested using trypsin (Sigma-Aldrich) after reaching ≥80% confluence and continuously propagated at 4,000 cells/cm^2 until reaching a senescent phase or passage (P). The senescent phase was defined as a decrease in the proliferative capacity ultimately leading to cell cycle arrest. BM-MSCs in the senescent phase were closely monitored for an additional 8 to 12 weeks before culture was interrupted to search for cells that had escaped senescence and recommenced proliferation. Post-senescence clones were isolated by a limiting dilution method: to obtain single-cell–derived clones, BM-MSCs were seeded at 1 cell/well in a 96-well culture plate (Corning Costar) and cultured as described above. We obtained 3 colonies. Split ratio (all of 3 colonies) is 1:4. The cells were observed daily for 4 to 6 weeks to examine colony formation. After the 2nd to 5th passages, flow cytometry was used to characterize BM-MSCs by detecting the expression of MSC

markers. The differentiation capacities of BM-MSCs were also proven through osteogenic and adipogenic differentiation. The high telomerase activity of BM-MSCs was measured by a telomerase activity assay. To generate BM-MSC-CM, BM-MSCs were grown to 80–90% confluence in 10 cm plate, washed thoroughly, and cultured in serum-free MEM (7 ml) for 24 h. BM-MSC-CM was collected by filtering through a 50-µm mesh. A human cytokine antibody array was used to measure the secretion of cytokines in control medium (Control) and human BM-MSC-CM (all of 3 colonies) to represent tumor-conditioned medium. All of the results were similar and represented in Supplementary Fig. 1e.

Human samples and IHC analysis

Sectioned human lung cancer specimens were obtained from GenDiscovery Biotechnology, Inc. All staining procedures were performed using a Super Sensitive IHC Detection Systems kit (BioGenex). Counterstaining was performed with haematoxylin. A semi-quantitative method for calculating positive signals was used. Signals were counted in six fields per sample under a light microscope at 400× magnification. The results were manually evaluated by two independent observers to determine both the percentage of positive cells and the staining intensity, as previously described [1-4]. The observers were blinded to the stage of each sample. The IHC score was obtained by multiplying the staining intensity (0 = no expression, 1 = weak expression, 2 = moderate expression, 3 = strong expression, and 4 = very strong expression) by the percentage of positive cells (0 = expression in 0–5% of cells, 1 = expression in 6–25% of cells, 2 = expression in 26–50% of cells, 3 = expression in 51–

75% of cells, and 4 = expression in 76–100% of cells) in the field. The maximum possible IHC score was 4 × 4 = 16.

Functional fractionation of cancer cells by invasion assays

Through four serial passages (p4), spheres derived from human lung cancer A549 cells were transferred back to adhesive tissue culture plates, after which they migrated back onto the plates and re-formed a monolayer with morphological heterogeneity [and were then collected as LM cells]. To establish an EMT/metastasis cell model, LM cells were seeded on Matrigel-coated Boyden chamber membranes. After a 24-hour incubation period, the cells that had invaded the Matrigel were collected as high-motility1 (HM1) cells, signifying one passage through the basement membrane matrix. Subsequently, these cells were re-cultured and passed 19 more times through the invasion–selection procedure (Fig 1c). The cells harvested from each subsequent round of selection were designated HM2 to HM20 cells [1].

ChIP

ChIP assays were performed as previously described [1-5]. Cells were fixed with 1% formaldehyde for 20 minutes at room temperature and harvested in ChIP lysis buffer (50 mM Tris (pH 8.0), 85 mM KCl, 1 mM DTT, 1 mM PMSF, 0.5% NP-40). Genomic DNA in the lysate was sonicated using a Bioruptor (Diagenode) for 15 cycles on a high power setting (30 s on, 30 s off) at 4°C. Cell debris was removed by centrifugation at 15,000 rpm for 30 minutes at 4°C. Lysate supernatants were brought up to a volume of 1 mL with ChIP lysis buffer and precleared using 5 μg of ssDNA and 25 μL of Protein

G agarose (ThermoFisher, Cat#20399) with rotation for 1 hour at 4°C. Precleared lysates were centrifuged at 14,000 rpm for 10 minutes, and lysate supernatants were transferred to new tubes for subsequent immunoprecipitation. A 50 μL volume of lysate was reserved as the whole-cell extract input control. Anti-GATA3 and STAT3 were used for immunoprecipitation. Antibodies were recovered using Protein G agarose (ThermoFisher). Immunoprecipitated DNA and input DNA were analysed by PCR using primers spanning the proximal (nucleotide positions -126/+164) promoter region of *PROM1*, the (-179/+139) promoter region of *ABCG1*, the (-276/-95) promoter region of *CDH1* or the (-32/+89) promoter region of *CDH1*. Following 30 cycles of amplification, PCR products were separated on a 1.5% agarose gel and analysed by ethidium bromide staining.

Luciferase reporter assay

The luciferase reporter plasmids were purchased from Addgene (Cat#12456 and Cat#12457). The luciferase reporter plasmids and their respective controls were introduced into cells. Cell lysates were harvested 24 hours later and analysed following the standard protocol of the dual luciferase reporter assay (Promega, Cat#RE1960). Luciferase activities were determined and are plotted as the fold change with overexpression or knockdown relative to the controls.

Western blotting

Western blotting was performed as previously described [1-4]. Images were acquired using a luminescence image analyser (FUSION SL; Vilber Lourmat), and band

intensities were quantitated by densitometry using Bio-1D and Bio-Gene software (Vilber Lourmat).

Sphere-forming culture

Spheres were generated as previously described [1-4]. Briefly, cells (1,000 cells/mL) were grown in suspension culture in ultra-low attachment plates (Corning) and serum-free RPMI medium (ATCC) supplemented with B27 (Invitrogen), 20 ng/mL EGF and 10 ng/mL bFGF (BD Biosciences). Spheres with a diameter of >30 μm were then counted. For serial passaging, spheres were harvested and dissociated to single cells with trypsin, and 100 dissociated cells were then re-plated in an ultra-low attachment 96-well plate and cultured for 12 days. Spheres were then re-counted. The individual spheres were found to be derived from single cells [6].

Microarray data collection and analysis

We obtained quantified time-course gene expression profiles for lung cancer cells cultured both with and without human BM-MSC-CM. Microarray profiles for the BM-MSC-treated and control cells were obtained at eight time points (2, 4, 8, 16, 24, 48, 72, and 96 hours post treatment) in triplicate samples. The microarray data were obtained with a Whole Genome OneArray® (HOA) and have been deposited in National Center for Biotechnology Information Gene Expression Omnibus (GEO). The flow chart and details of our method are summarized in Supplementary information and Supplementary Fig. 4.

QPCR

QPCR was used to quantify gene expression levels. Gene expression analysis was conducted with SYBR Green (Thermo Fisher Scientific, Waltham, MA, USA) on a Step One Real Time PCR System (Applied Biosystems). Primers used for Q-PCR are as follows: NANOG: F 5′-ctccaacatcctgaacctcagc-3′ R 5′-cgtcacaccattgctattcttcg-3′; ABCB1: F 5′-gctgtcaaggaagccaatgcct-3′ R 5′-tgcaatggcgatcctctgcttc-3′; ABCC2: F 5′-gccaacttgtggctgtgatagg-3′ R 5′-atccaggactgctgtgggacat-3′; ABCG1: F 5′-gagggatttgggtctgaactgc-3′ R 5′-tctcaccagccgactgttctga-3′; TWIST1: F 5′-gccaggtacatcgacttcctct-3′ R 5′-tccatcctccagaccgagaagg-3′; VIM: F 5′-aggcaaagcaggagtccactga-3′ R 5′-atctggcgttccagggactcat-3′; GAPDH: F 5′-gtctcctctgacttcaacagcg-3′ R 5′-accaccctgttgctgtagccaa-3′; IL6: F 5′-agacagccactcacctcttcag-3′ R 5′-ttctgccagtgcctctttgctg-3′; IL6R: F 5′-gactgtgcacttgctggtggat-3′ R 5′-acttcctcaccaagagcacagc-3′; LIF: F 5′-agatcaggagccaactggcaca-3′ R 5′-gccacatagcttgtccaggttg-3′; LIFR: F 5′-caccttccaaaatagcgagtatgg-3′ R 5′-atggttccgaccgagacgagtt-3′; GATA3: F 5′-accacaaccacactctggagga-3′ R 5′-tcggtttctggtctggatgcct-3′; CCND1: F 5′-tctacaccgacaactccatccg-3′ R 5′-tctggcattttggagaggaagtg-3′; CDH1: F 5′-gcctcctgaaaagagagtggaag-3′ R 5′-tggcagtgtctctccaaatccg-3′; PROM1: F 5′-cactaccaaggacaaggcgttc-3′ R 5′-caacgcctctttggtctccttg-3′; POU5F1: F 5′-cctgaagcagaagaggatcacc-3′ R 5′-aaagcggcagatggtcgtttgg-3′; and SOX2: F 5′-gctacagcatgatgcaggacca-3′ R 5′-tctgcgagctggtcatggagtt-3′.

Cytokine array

Detection of cytokines, chemokines, and growth factors released in BM-MSC-CM was done using the human Cytokine Array C5 (AAH-CYT-5-4) following the manufacturer's instructions (RayBiotech, Norcross, GA, USA). Densitometry analyses were done using the Fluor Chem FC3 System imaging apparatus (ProteinSimple Co., Santa Clara, CA, USA) with AlphaView Software (ProteinSimple). All values were normalized with the mean intensity of the positive and negative controls.

Animal experiments

Female CB17 severe combined immunodeficiency (SCID) mice (6 weeks old) were used, and all experimental protocols were approved by the Animal Studies Committee of National Tsing Hua University.

The bone homing assay

2×10^5 (1×10^5 LM and 1×10^5 HM20 cells) or 3×10^5 (the above cells co-cultured with 1×10^5 BM-MSCs) cells were cultured under sphere-forming conditions for 7 days before injection (in a volume of exactly 100 μL) into the left cardiac ventricle of the 6-week-old mice. Mice were sacrificed on day 5 after intracardiac inoculation. The hind limbs were flushed, and cells were isolated and cultured [7].

An experimental model of bone metastasis by human lung cancer cells

For subcutaneously tumor growth, mice (n = 1) were injected subcutaneously with 1 x 10^6 A549 cells in 100 uL of a 1:1 mixture of DMEM/Matrigel. Tumorigenicity was

evaluated at 4 weeks after transplantation. For lung orthotopic injection [8], tumor fragments (1 mm^3) derived from the A549 subcutaneously tumor growing in a mixture of DMEM/Matrigel (1:1) were implanted by lung orthotopic injection. The mice were anesthetized by isofluran inhalation. A 0.8-cm transverse incision of skin was made in the left chest wall. A 0.4-0.5 cm intercostal incision between the third and fourth rib on the chest wall was made, and the chest wall was opened. The left lung was taken up by a forceps, and tumor fragments were sewn promptly into the upper lung by one suture. The lung was then returned into the chest cavity and the incision in the chest wall was closed with a 6-0 surgical suture. After closing the chest wall, an intrathoracic puncture was made by using a 3-ml syringe and 25-gauge 1/2 needle to withdraw the remaining air in the chest cavity. Then the skin and chest muscle were closed with a 6-0 surgical suture in one layer. For MSC recruitment assay [9], 0.5×10^6 MSC-RFP cells were injected intravenously after lung orthotopic injection for 1 week (n = 3). The primary lung tumors were harvested after 5 days for FACS analysis of MSC-RFP cells. For analysis of bone metastases. SCDCs were isolated from long bones after for FACS analysis of various cancer cells after MSC-RFP cells injection for 1 (n = 3) and 2 weeks (n = 3). The performance status of the mice began to decrease, at which time the animals were sacrificed and autopsied. The orthotopic primary tumor and all major organs as well as the whole skeleton were explored (n = 5). No randomization is necessary and no blinding was done for this study.

Isolation of SCDCs from long bones

Metastatic cells were isolated from the BM of the lower limbs. Long bones were excised and cleaned of all soft tissues. Marrow cells were released by "flushing," in which 5 to

10 mL of α-MEM containing 2 × penicillin/streptomycin was introduced via a 27-gauge needle into the distal epiphysis through the BM compartment. Cell clumps were disaggregated by passing medium containing cells through a 27-gauge needle syringe. Cells were plated in 10-cm dishes and expanded for 5 days in medium containing 0.6 mg/mL G-418. This procedure was conducted separately for each femur and tibia from 5 mice per group. SCDCs were counted under a light microscope after crystal violet staining [7].

Data source

Collection of MSC conditioned medium human bone marrow-mesenchymal stem cells (BM-MSCs) were seeded in a 10 cm plate with 7 ml growth medium and then cultured for 72 hours. After 72 hours, the medium was then collected, filtered and stored at −20 ℃. We obtained quantified time-course gene expression profiles for A549 lung cancer cells both with and without BM-MSC-conditional medium (BM-MSCs-CM). The MSCs-treated and control microarray profiles were observed at eight time-points (2, 4, 8, 16, 24, 48, 72, and 96 hours post-treatment) using three replicates. The microarray data were provided by Whole Genome OneArray®(HOA) and have been deposited in National Center for Biotechnology Information Gene Expression Omnibus (GEO).

Microarray analysis

Total RNA was extracted using the TRIzol® Reagent (Invitrogen,Carlsbad, CA) according to the manufacturer's instructions. The concentration and purity of the RNA was measured by NanoDrop 1000 spectrophotometer (Thermo Fisher Scientific). Purity was checked by the ratio of the OD260/OD280 and OD260/OD230. The quality of total

RNA was accessed using Agilent 2100 Bioanalyzer (Agilent Technologies, Santa Clara, CA, USA)

Fluorescent RNA targets were prepared from 1 μg total RNA samples using OneArray® Amino Allyl aRNA Amplification Kit (Phalanx Biotech Group, Taiwan) and Cy5 dye (GE Healthcare). Fluorescent targets were hybridized to the Human Whole Genome OneArray® with Phalanx hybridization buffer using Phalanx Hybridization System. After 16 hrs hybridization, non-specific binding targets were washed away. The slides scanned using a DNA Microarray Scanner (Model G2505C, Agilent Technologies). The Cy5 fluorescent intensities of each spot were analyzed by GenePix 4.1 software (Molecular Devices).

Each single sample was at least performed twice in terms of technical or biological replicates under the reproducibility more than 0.975. The signal intensity was loaded into Rosetta Resolver System® (Rosetta Biosoftware, USA) to do data preprocessing and apply to 75 percentile centering normalization. The errors of the sample were estimated by using the error-weighted approach at the same time. Both of fold change and p-value for pair-wise sample comparison were calculated for evaluating differentially expressed genes. The criteria with fold change ≥ 2 or ≤ -2 and p-value < 0.05 are strongly recommended for further analysis.

Target protein pool determination

In our proposed approach, two types of data are needed for the construction of MSCs and Control PPI networks: (i) the protein-protein interactions (PPI) data of Homo sapiens, and (ii) two kinds of time-series microarray profiles. The PPI data of Home

sapiens were obtained from Biological General Repository for Interaction Datasets (BioGRID) database (http://thebiogrid.org/) [9]. For both time-series microarray data, one way of analysis of variance (ANOVA) was applied to select proteins having significant variation with the null hypothesis being that all protein profiles are the same within the eight time-points. For our proposed approach, we rejected the null hypothesis when the p-value, adjusted by Bonferroni, is less than 0.05. Proteins thus selected are included in our protein pools for both sets of microarray data. After the protein pools were determined, two putative PPI networks were constructed by connecting the proteins in our protein pools using all available protein interactions from BioGRID.

Construction of MSCs-treated and Control PPI network

From the above-mentioned process, we have selected the protein pools and constructed two putative PPI networks. Putative PPI networks, including all available protein interactions under different conditions and literatures from BioGRID, were further refined with the MSCs-treated and Control microarray to remove false-positive interactions. In order to remove the false positive PPIs, the putative interactions were pruned through the dynamic PPI model, Akaike information Criterion (AIC), and Student's t-test. Following are descriptions of these proposed approaches. For the k-th target protein in the intracellular PPI network, the dynamic PPI model can be described as the following:

$$x_k(t+1) = x_k(t) - d_k x_k(t) + \sum_{i=1}^{N_k} \alpha_{ki}\, x_i(t) + b_k + n_k(t) \qquad [1]$$

where $x_k(t)$ represents the protein activity level for k-th target protein at time t, $d_k$represents the degradation for k-th target protein, $x_i(t)$ denotes the protein activity level for i-th protein interacting with k-th target protein, and α_{ki} denotes the interaction ability between the k-th target protein and i-th protein interacting with k-th target protein. We denote that N_k is the number of protein-protein interaction that interacts with the k-th target protein via BioGRID database. The basal level of k-th target protein is denoted by b_k, and the stochastic noise attributed to model uncertainty and other deviant factors is denoted by $n_k(\mathrm{t})$.

In order to determine the interaction ability, degradation, and basal level, it is necessary to exploit the time-series microarray data and PPI information. To identify these parameters in this model, we replace the all protein activity levels with the gene expression profiles. The dynamic PPI model in equation [1] can be rewritten as the following:

$$x_k(t+1) = [x_1(t) \quad x_2(t) \quad x_3(t) \cdots x_{N_k}(t) \quad x_k(t) \quad 1] \cdot \begin{bmatrix} \alpha_{k1} \\ \alpha_{k2} \\ \alpha_{k3} \\ \vdots \\ \alpha_{kN_k} \\ 1-d_k \\ b_k \end{bmatrix} \qquad [2]$$

$$+ n_k(t) \; = \psi_k[t]\eta_k + n_k(t)$$

where $\psi_k[t]$represents the regression vector and $\eta_k$represents the interaction parameter vector which is necessary to be determined for the target k-th protein. In order to avoid overfitting in the procedure for estimated parameters, we exploited the cubic spline method to interpolate extra time-points within the eight time-points (L, total number of time-points, amounts five times the number of elements in interaction parameter

vectors). After the interpolation, the equation [2] for different time points can further be rewritten as follows:

$$\begin{bmatrix} x_k(t_2) \\ x_k(t_3) \\ \vdots \\ x_k(t_L) \end{bmatrix} = \begin{bmatrix} \psi_k(t_1) \\ \psi_k(t_2) \\ \vdots \\ \psi_k(t_{L-1}) \end{bmatrix} \eta_k + \begin{bmatrix} n_k(t_1) \\ n_k(t_2) \\ \vdots \\ n_k(t_{L-1}) \end{bmatrix} \qquad [3]$$

We defined that $x_k = [x_k(t_2) \quad \cdots \quad x_k(t_L)]^T$, $\psi_k = [\psi_k(t_1) \quad \cdots \quad \psi_k(t_{L-1})]^T$ and $n_k = [n_k(t_1) \quad \cdots \quad n_k(t_{L-1})]^T$. Hence, equation [3] can be rewritten in the following form:

$$x_k = \psi_k \eta_k + n_k \qquad [4]$$

For the interaction parameter vector, we assumed that the basal level b_k should be equal or greater than zero for each target protein. For [4], the constrained least-squares minimization algorithm could be exploited to identify the interaction parameter vector [10]. Therefore, the least-squares minimization equation and the assumption can be shown in the following form:

$$\min \frac{1}{2} \|\psi_k \eta_k - x_k\|_2^2, s.t. A\eta_k \leq 0 \qquad [5]$$

where A= $[0 \quad 0 \quad \cdots \quad -1]$. After the interaction parameter vectors for all proteins in the putative PPI networks were identified, the parameter vectors would further be estimated and be used to select significant interactions through Akaike's Information Criterion (AIC) so as to removing the false positive PPIs. AIC method can be shown as the following:

$$\mathrm{AIC}(N_k) = \log \epsilon_k + \frac{2N_k}{L} \qquad [6]$$

where $\varepsilon_k = \frac{1}{L}(x_k - \hat{x}_k)^T(x_k - \hat{x}_k)$ is the estimated residual error, N_k represents the number of elements in interaction parameter vectors, and L represents the total number of interpolated time-points samples used to estimate the interaction parameter vectors. The AIC value is comprised of the estimated residual error and model complexity. The AIC value increases as the estimated residual error increases. In other words, the AIC value decreases as the number of parameters decreases. Therefore, the AIC provides a way to achieve a good tradeoff between estimated residual error and model complexity. We obtain the number of interaction parameters when the AIC value is minimal for the target k-th protein. After the parameter vector is estimated through the AIC method, the student's t-test is further used to determine whether the interaction parameters are significant. The null hypothesis of the student's test is that each of interaction parameters is zero ($\alpha_{ki} = 0$), and then we rejected the null hypothesis when the p-value, adjusted by Bonferroni, is equal to or less than 0.05. The flow chart of our method is summarized in Supplementary Fig. 4.

References

1 Chang YW, Su YJ, Hsiao M, Wei KC, Lin WH, Liang CL, et al. Diverse Targets of beta-Catenin during the Epithelial-Mesenchymal Transition Define Cancer Stem Cells and Predict Disease Relapse. Cancer Res. 2015;75:3398-410.

2 Su YJ, Chang YW, Lin WH, Liang CL, Lee JL. An aberrant nuclear localization of E-cadherin is a potent inhibitor of Wnt/beta-catenin-elicited promotion of the cancer stem cell phenotype. Oncogenesis. 2015;4:e157.

3 Su YJ, Lai HM, Chang YW, Chen GY, Lee JL. Direct reprogramming of stem cell properties in colon cancer cells by CD44. EMBO J. 2011;30:3186-99.

4 Su YJ, Lin WH, Chang YW, Wei KC, Liang CL, Chen SC, et al. Polarized cell migration induces cancer type-specific

CD133/integrin/Src/Akt/GSK3beta/beta-catenin signaling required for maintenance of cancer stem cell properties. Oncotarget. 2015;6:38029-45.

5 Lee JL, Wang MJ, Chen JY. Acetylation and activation of STAT3 mediated by nuclear translocation of CD44. J Cell Biol. 2009;185:949-57.

6 Mani SA, Guo W, Liao MJ, Eaton EN, Ayyanan A, Zhou AY, et al. The epithelial-mesenchymal transition generates cells with properties of stem cells. Cell. 2008;133:704-15.

7 Valencia K, Ormazabal C, Zandueta C, Luis-Ravelo D, Anton I, Pajares MJ, et al. Inhibition of collagen receptor discoidin domain receptor-1 (DDR1) reduces cell survival, homing, and colonization in lung cancer bone metastasis. Clin Cancer Res. 2012;18:969-80.

8 Yang M, Hasegawa S, Jiang P, Wang X, Tan Y, Chishima T, et al. Widespread skeletal metastatic potential of human lung cancer revealed by green fluorescent protein expression. Cancer Res. 1998;58:4217-21.

9 Chaturvedi P, Gilkes DM, Wong CC, Luo W, Zhang H, Wei H, et al. Hypoxia-inducible factor-dependent breast cancer-mesenchymal stem cell bidirectional signaling promotes metastasis. J Clin Invest. 2013;123:189-205.

10 Wang YC, Chen BS. Integrated cellular network of transcription regulations and protein-protein interactions. BMC Syst Biol. 2010;4:20.
